# Retraction: MiRNA-107 enhances the malignant progression of pancreatic cancer by targeting TGFBR3

**DOI:** 10.1371/journal.pone.0263037

**Published:** 2022-01-21

**Authors:** The PLOS ONE Editors

After this article was published, similarities were noted between this article and submissions by other research groups which call into question the validity and provenance of the reported results and the adherence of this article to the PLOS Authorship policy. The authors provided data files to support the article’s results, but the comments and files received did not resolve the concerns.

In discussing this matter, the corresponding author requested the article’s retraction.

In light of the above issues, the *PLOS ONE* Editors retract this article [1].

JC agreed with retraction. TT, QY, CZ, and XL did not respond or could not be reached.
